# Supplementary material for: Reverse Genetic Analysis of the Yeast RSC Chromatin Remodeler Reveals a Role for RSC3 and SNF5 Homolog 1 in Ploidy Maintenance
Source: PLoS Genet. 2007 Jun 1;3(6):e92. doi: 10.1371/journal.pgen.0030092 (PMC1885278; doi:10.1371/journal.pgen.0030092)
Supplement: Supplementary file 4 [file pgen.0030092.sg004.pdf]

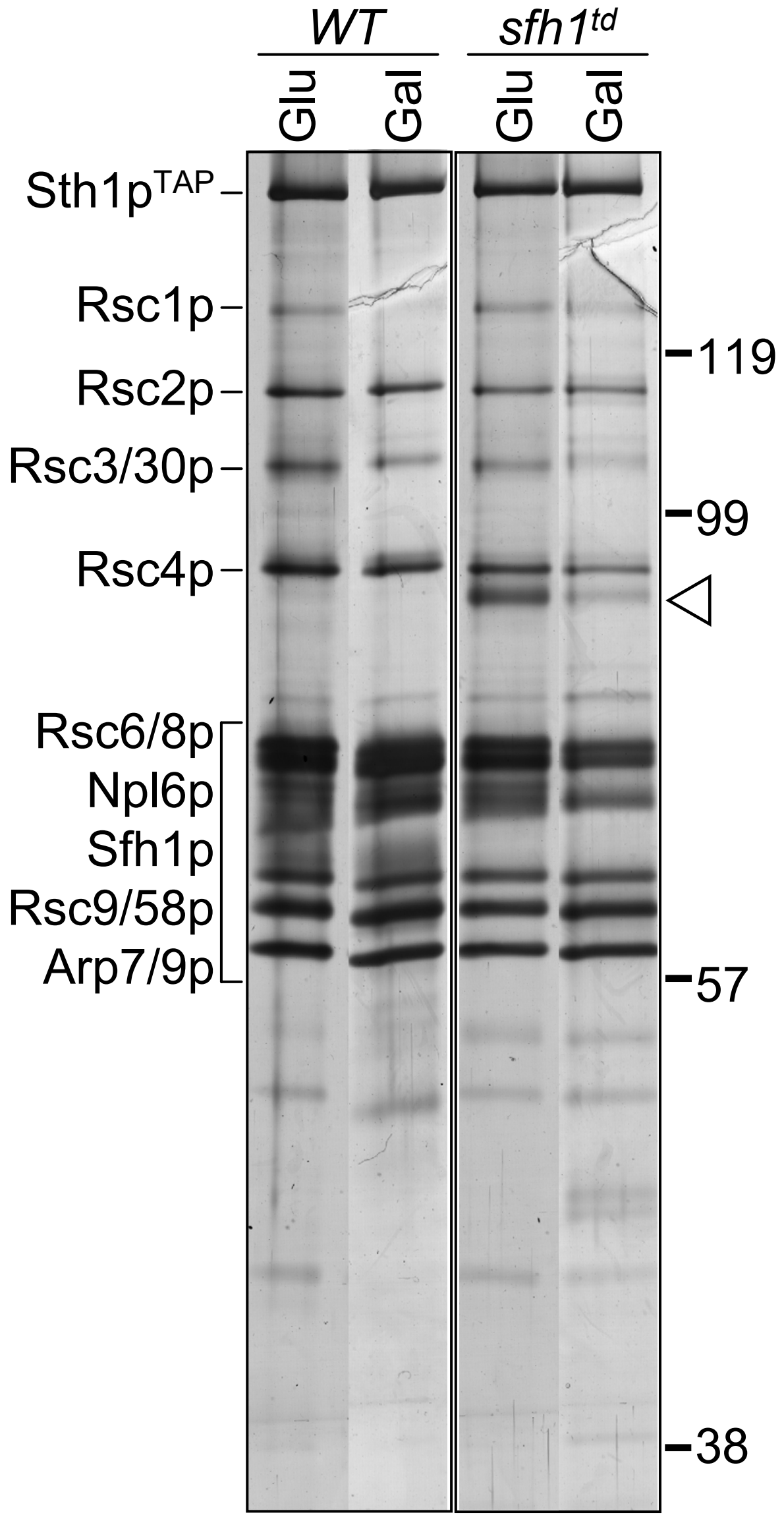

Campsteijn *et al*, Supplemental Figure 4
