# Supplementary material for: Reverse Genetic Analysis of the Yeast RSC Chromatin Remodeler Reveals a Role for RSC3 and SNF5 Homolog 1 in Ploidy Maintenance
Source: PLoS Genet. 2007 Jun 1;3(6):e92. doi: 10.1371/journal.pgen.0030092 (PMC1885278; doi:10.1371/journal.pgen.0030092)
Supplement: Supplementary file 6 [file pgen.0030092.st001.doc]

| ***rsc3^td^*** | ***rsc4^td^*** | ***rsc6^td^*** | ***rsc8^td^*** | ***rsc9^td^*** | ***rsc58^td^*** | ***sfh1^td^*** |  |
| --- | --- | --- | --- | --- | --- | --- | --- |
| + | + | + | + | + | NT | + | ***sth1^td^*** |
|  | + | + | + | + | + | ― | ***rsc3^td^*** |
|  |  | + | NT | + | NT | + | ***rsc4^td^*** |
|  |  |  | + | + | NT | + | ***rsc6^td^*** |
|  |  |  |  | + | + | + | ***rsc8^td^*** |
|  |  |  |  |  | + | + | ***rsc9^td^*** |
|  |  |  |  |  |  | + | ***rsc58^td^*** |
