# Supplementary material for: Reverse Genetic Analysis of the Yeast RSC Chromatin Remodeler Reveals a Role for RSC3 and SNF5 Homolog 1 in Ploidy Maintenance
Source: PLoS Genet. 2007 Jun 1;3(6):e92. doi: 10.1371/journal.pgen.0030092 (PMC1885278; doi:10.1371/journal.pgen.0030092)
Supplement: Supplementary file 7 [file pgen.0030092.st002.doc]

| Strain | Genotype | Source |
| --- | --- | --- |
| YN2 | MATa; trp1-1; ura3-1; his3-11;15; leu2-3;112 | A. Tzalagoff |
| YN 18 | *MAT*a; *ade2-1; can1-100; his3-11,15; leu2-3, 112; trp1-1; ura3-1; Δlys2::rKWD50N* | M. Gartenberg |
| YN 94 | *MAT*a*; P_gal1-10_-myc::UBR1-HIS3; trp1-1; ura3,1; his3-11,15; leu2-3,112* | This study |
| YN 106 | *MAT*a**/**α*; UBR1/P_gal1-10_-myc::UBR1-HIS3* | This study |
| YN 119 | *MAT*a; *P_gal1-10_::UBR1-HIS3; P_cup1_-degron::sth1-URA3* | This study |
| YN 146 | *MAT*a; *P_gal1-10_::UBR1-HIS3; P_cup1_-degron::sth1-URA3; P_cup1_-degron::rsc8-TRP1* | This study |
| YN 147 | *MAT*a; *P_gal1-10_::UBR1-HIS3; P_cup1_-degron::rsc3-URA3* | This study |
| YN 148 | *MAT*a; *UBR1; P_cup1_-degron::rsc3-URA3* | This study |
| YN 158 | *MAT*α*;P_gal1-10_::UBR1-HIS3; P_cup1_-degron::rsc8-URA3* | This study |
| YN 159 | *MAT*a*;* *P_gal1-10_::UBR1-HIS3; P_cup1_-degron::rsc8-URA3* | This study |
| YN 182 | *MAT*a; *P_gal1-10_::UBR1-HIS3; P_cup1_-degron::rsc8-TRP1* | This study |
| YN 184 | *MAT*α*; P_gal1-10_::UBR1-HIS3; P_cup1_-degron::rsc8-TRP1* | This study |
| YN 191 | *MAT*a; *P_gal1-10_::UBR1-HIS3; P_cup1_-degron::sth1-TRP1* | This study |
| YN 218 | *MAT*a; *P_gal1-10_::UBR1-HIS3; P_cup1_-degron::rsc4-URA3* | This study |
| YN 220 | *MAT*a; *P_gal1-10_::UBR1-HIS3; P_cup1_-degron::sfh1-TRP1* | This study |
| YN 223 | *MAT*a; *P_gal1-10_::UBR1-HIS3; P_cup1_-degron::cdc15-TRP1* | This study |
| YN 229 | *MAT*α; *P_gal1-10_::UBR1-HIS3; P_cup1_-degron::sth1-LEU2* | This study |
| YN 232 | *MAT*α; *P_gal1-10_::UBR1-HIS3; P_cup1_-degron::rsc6-LEU2* | This study |
| YN 248 | *MAT*α; *P_gal1-10_::UBR1-HIS3; P_cup1_-degron::sfh1-LEU2* | This study |
| YN 251 | *MAT*a; *P_gal1-10_::UBR1-HIS3; P_cup1_-degron::rsc8-TRP1; P_cup1_-degron::rsc3-URA3* | This study |
| YN 262 | *MAT*α; *P_gal1-10_::UBR1-HIS3; P_cup1_-degron::rsc9-TRP1* | This study |
| YN 263 | *MAT*α; *P_gal1-10_::UBR1-HIS3; P_cup1_-degron::rsc58-TRP1* | This study |
| YN 272 | *MAT*a; *P_gal1-10_::UBR1-HIS3; P_cup1_-degron::sth1-TRP1; SWE1:: P_gal1-10_::SWE1-URA3* | This study |
| YN 286 | *MAT*a*; P_gal1-10_::UBR1-HIS3; P_cup1_-degron::sth1-URA3 P_cup1_-degron::rsc8-TRP1; P_cup1_-degron::rsc6-LEU2* | This study |
| YN 290 | *MAT*a*; P_gal1-10_::UBR1-HIS3; P_cup1_-degron::sth1-URA3 P_cup1_-degron::rsc6-LEU2* | This study |
| YN 291 | *MAT*α*; P_gal1-10_::UBR1-HIS3; P_cup1_-degron::sth1-URA3 P_cup1_-degron::rsc6-LEU2* | This study |
| YN 294 | *MAT*a*;* *P_gal1-10_::UBR1-HIS3; P_cup1_-degron::rsc8-URA3; P_cup1_-degron::sfh1-TRP1* | This study |
| YN 299 | *MAT*α; *P_gal1-10_::UBR1-HIS3; P_cup1_-degron::rsc8-URA3; P_cup1_-degron::rsc6-LEU2* | This study |
| YN 315 | *MAT*a; *P_gal1-10_::UBR1-HIS3; P_cup1_-degron::rsc58-TRP1* | This study |
| YN 317 | *MAT*a*; P_gal1-10_::UBR1-HIS3; P_cup1_-degron::rsc8-URA3; P_cup1_-degron::rsc58-TRP1* | This study |
| YN 319 | *MAT*a; *P_gal1-10_::UBR1-HIS3; P_cup1_-degron::rsc6-LEU2* | This study |
| YN 321 | *MAT*a*; P_gal1-10_::UBR1-HIS3; P_cup1_-degron::rsc9-TRP1* | This study |
| YN 324 | *MAT*a*; P_gal1-10_::UBR1-HIS3; P_cup1_-degron::rsc9-TRP1; P_cup1_-degron::rsc8-URA3* | This study |
| YN 326 | *MAT*a;*; P_gal1-10_::UBR1-HIS3; P_cup1_-degron::rsc9-TRP1; P_cup1_-degron::rsc3-URA3* | This study |
| YN 338 | *MAT*α; *UBR1; P_cup1_-degron::sfh1-LEU2* | This study |
| YN344 | *MAT*a; *P_gal1-10_::UBR1-HIS3; P_cup1_-degron::rsc6-LEU2; P_cup1_-degron::rsc3-URA3* | This study |
| YN 351 | *MAT*α; *P_gal1-10_::UBR1-HIS3; P_cup1_-degron::sth1-LEU2; P_cup1_-degron::rsc3-URA3* | This study |
| YN 358 | *MAT*a*; P_gal1-10_::UBR1-HIS3; P_cup1_-degron::sfh1-TRP1; P_cup1_-degron::sth1-URA3* | This study |
| YN 362 | *MAT*a*; P_gal1-10_::UBR1-HIS3; P_cup1_-degron::sfh1-TRP1; P_cup1_-degron::rsc6-LEU2* | This study |
| YN 363 | *MAT*a*; P_gal1-10_::UBR1-HIS3; P_cup1_-degron::rsc9-TRP1; P_cup1_-degron::sfh1-LEU2* | This study |
| YN 365 | *MAT*a*; P_gal1-10_::UBR1-HIS3; P_cup1_-degron::rsc4-URA3; P_cup1_-degron::sfh1-LEU2* | This study |
| YN 367 | *MAT*a*; P_gal1-10_::UBR1-HIS3; P_cup1_-degron::cdc20-URA3* | This study |
| YN 372 | *MAT*α; *P_gal1-10_::UBR1-HIS3; P_cup1_-degron::rsc3-LEU2* | This study |
| YN 381 | *MAT*a*; P_gal1-10_::UBR1-HIS3; P_cup1_-degron::cdc20-URA3; P_cup1_-degron::sth1-LEU2* | This study |
| YN 388 | *MAT*α*; P_gal1-10_::UBR1-HIS3; P_cup1_-degron::sfh1-TRP1; P_cup1_-degron::rsc6-LEU2; P_cup1_-degron::sth1-URA3* | This study |
| YN 400 | *MAT*α; *P_gal1-10_::UBR1-HIS3; sth1::TAPtag-URA3* | This study |
| YN438 | *MAT*a*; P_gal1-10_::UBR1-HIS3; sth1::TAPtag-TRP1; P_cup1_-degron::rsc8-URA3* | This study |
| YN 453 | *MAT*a*; P_gal1-10_::UBR1-HIS3; sth1::TAPtag-TRP1; P_cup1_-degron::sfh1-LEU2* | This study |
| YN 520 | *MAT*a*; P_gal1-10_::UBR1-HIS3; SWE1:: P_gal1-10_::SWE1-URA3* | This study |
| YN 594 | *MAT*a*; P_gal1-10_::UBR1-HIS3; P_cup1_-degron::cdc15-TRP1; P_cup1_-degron::sth1-LEU2* | This study |
| YN 671 | *MAT*α*; P_gal1-10_::UBR1-HIS3; P_cup1_-degron::rsc6-LEU2; P_cup1_-degron::rsc9-TRP1* | This study |
| YN 908 | *MAT*a*; P_gal1-10_::UBR1-HIS3; P_cup1_-degron::rsc4-URA3; P_cup1_-degron::rsc9-TRP1* | This study |
| YN 995 | *MAT*α*; P_gal1-10_::UBR1-HIS3; P_cup1_-degron::rsc58-TRP1; P_cup1_-degron::sfh1-LEU2* | This study |
| YN 1098 | *MAT*α/α*; P_gal1-10_::UBR1-HIS3/ P_gal1-10_::UBR1-HIS3; P_cup1_-degron::rsc3-URA3/P_cup1_-degron::rsc3-URA3; P_cup1_-degron::sfh1-LEU2/ P_cup1_-degron::sfh1-LEU2* | This study |
| YN 1106 | *MAT*a/a*; P_gal1-10_::UBR1-HIS3/ P_gal1-10_::UBR1-HIS3; P_cup1_-degron::rsc3-URA3/P_cup1_-degron::rsc3-URA3; P_cup1_-degron::sfh1-LEU2/ P_cup1_-degron::sfh1-LEU2; P_cup1_-degron::rsc9-TRP1/ P_cup1_-degron::rsc9-TRP1* | This study |
| YN 1438 | *MAT*α/α/a/a*; P_gal1-10_::UBR1-HIS3/ P_gal1-10_::UBR1-HIS3/ P_gal1-10_::UBR1-HIS3/ P_gal1-10_::UBR1-HIS3; rsc3/rsc3/P_cup1_-degron::rsc3-URA3/P_cup1_-degron::rsc3-URA3; P_cup1_-degron::sfh1-LEU2/ P_cup1_-degron::sfh1-LEU2/ P_cup1_-degron::sfh1-LEU2/ P_cup1_-degron::sfh1-LEU2; rsc9/rsc9/ P_cup1_-degron::rsc9-TRP1/ P_cup1_-degron::rsc9-TRP* | This study |
| YN 1450 | *MAT*a; *ade2-1; can1-100; leu2-3; 112; his3-11; 15; ura3-1; cdc6::hisG-URA3-hisG;*  *trp1-1::TRP1-P_met3_-cdc6* | Nasmyth K3596 |
| YN 1453 | *MAT*a; *ade2-1; trp1-1; can1-100; leu2-3, 112; his3-11, 15; ura3; clb5::URA3* | Nasmyth K3095 |
|  | *MAT*α*; his3Δ1; leu2Δ0; lys2Δ0; ura3Δ0; mbp1::kanMX4* | Euroscarf Y13753 |
|  | *MAT*a*; his3Δ1; leu2Δ0; lys2Δ0; ura3Δ0; cln1::kanMX4* | Euroscarf Y00785 |
|  | *MAT*α*; his3Δ1; leu2Δ0; lys2Δ0; ura3Δ0; cln1::kanMX4* | Euroscarf Y10785 |
|  | *MAT*a*; his3Δ1; leu2Δ0; lys2Δ0; ura3Δ0; cln2::kanMX4* | Euroscarf Y01036 |
|  | *MAT*α*; his3Δ1; leu2Δ0; lys2Δ0; ura3Δ0; cln2::kanMX4* | Euroscarf Y11036 |
|  | *MAT*a*; his3Δ1; leu2Δ0; lys2Δ0; ura3Δ0; cln3::kanMX4* | Euroscarf Y00366 |
|  | *MAT*α*; his3Δ1; leu2Δ0; lys2Δ0; ura3Δ0; cln3::kanMX4* | Euroscarf Y10366 |
|  | *MAT*a*; his3Δ1; leu2Δ0; lys2Δ0; ura3Δ0; clb1::kanMX4* | Euroscarf Y04738 |
|  | *MAT*α*; his3Δ1; leu2Δ0; lys2Δ0; ura3Δ0; clb1::kanMX4* | Euroscarf Y14738 |
|  | *MAT*a*; his3Δ1; leu2Δ0; lys2Δ0; ura3Δ0; clb2::kanMX4* | Euroscarf Y05534 |
|  | *MAT*α*; his3Δ1; leu2Δ0; lys2Δ0; ura3Δ0; clb2::kanMX4* | Euroscarf Y15534 |
|  | *MAT*a*; his3Δ1; leu2Δ0; lys2Δ0; ura3Δ0; clb3::kanMX4* | Euroscarf Y03853 |
|  | *MAT*α*; his3Δ1; leu2Δ0; lys2Δ0; ura3Δ0; clb3::kanMX4* | Euroscarf Y13853 |
|  | *MAT*a*; his3Δ1; leu2Δ0; lys2Δ0; ura3Δ0; clb4::kanMX4* | Euroscarf Y04159 |
|  | *MAT*α*; his3Δ1; leu2Δ0; lys2Δ0; ura3Δ0; clb4::kanMX4* | Euroscarf Y14159 |
|  | *MAT*a*; his3Δ1; leu2Δ0; lys2Δ0; ura3Δ0; clb5::kanMX4* | Euroscarf Y05535 |
|  | *MAT*α*; his3Δ1; leu2Δ0; lys2Δ0; ura3Δ0; clb5::kanMX4* | Euroscarf Y15535 |
|  | *MAT*a*; his3Δ1; leu2Δ0; lys2Δ0; ura3Δ0; clb6::kanMX4* | Euroscarf Y04739 |
|  | *MAT*α*; his3Δ1; leu2Δ0; lys2Δ0; ura3Δ0; clb6::kanMX4* | Euroscarf Y14739 |

Campsteijn *et al*, Supplemental Table 2
